# Accuracy of intraocular lens power calculation in high myopia

**DOI:** 10.4103/0974-620X.71888

**Published:** 2010

**Authors:** Asaad A. Ghanem, Hosam M. El-Sayed

**Affiliations:** Ophthalmology Center, Faculty of Medicine, Mansoua University, Mansoura, Egypt

**Keywords:** High myopia, intraocular lens power calculation, phacoemulsification

## Abstract

**Purpose:**

To study the accuracy of different recent intraocular lens (IOL) calculation formulas in predicting a target postoperative refraction ± 1.0*D* (Diopters) in patients with long eyes (axial length ≥ 26.0 mm) undergoing phacoemulsification.

**Materials and Methods:**

This study comprised 127 eyes of 87 patients who presented with cataract and axial eye length ≥ 26 mm. Before phacoemulsification and IOL implantation; axial length measurement using immersion ultrasound A-scan technique, and autokeratometry with or without computerized corneal topography for K readings were done. The IOL power was calculated using four formulas, namely the SRK-T, Hoffer-Q, Holladay-2, and Haigis formulas. Four months after surgery, refraction was done. Differences between actual postoperative refraction and assumed target refraction using the different formulas were analyzed. *P* < 0.05 was considered statistically significant.

**Results:**

In all 127 eyes, the mean axial length was 31.71 mm (range, 26.06–37.11 mm) and the mean K was 44.68 *D* (range, 40.05–55.14*D*). The mean preoperative spherical equivalent (SE) was −17.52D (range, −12.25 to −30.50*D*). After surgery, the mean spherical equivalent was −0.8 ± 0.83*D* (range, +1.25 to −3.75*D*). The mean postoperative refractive SE when implanting a plus power IOLs was −0.3 ± 0.51*D* (*P* < 0.001) while the mean postoperative refractive SE when implanting a minus power IOLs was +1.21 ± 0.11*D* denoting a highly significant tendency toward hyperopia (*P* < 0.001). Concerning the minus power group, most postoperative refractive error was within +1.0 to +2.0*D* in the actual implanted IOL and in all other formula calculated IOL power. However, Haigis formula showed the least deviation while SRK-T and other formulas showed a greater tendency toward hyperopia.

**Conclusions:**

In eyes with high axial myopia, the performance of SRK-T, Hoffer-Q, Holladay-2 and Haigis formulas are comparable in low plus-powered IOL implantation. Haigis formula is the best formula when minus power IOL is implanted.

## Introduction

In extreme myopia, implantation of a weak or even plano posterior chamber intraocular lens (IOL) is considered preferable to aphakia, because it stabilizes the vitreous base and reduces complications associated with posterior capsule opacification.[1]

One of the final frontiers in ophthalmology is consistent accurate estimation of IOL powers over a wide range of axial lengths. It is generally accepted that both theoretical and regression IOL formulas perform well for eyes of average axial (22.0–24.5 mm).[2] However, *precise* biometry predication in extremely long eyes requiring concave lenses has always been *difficult*.[3–5]

The aim of the study was to evaluate the performance of different recent IOL power calculation formulas (SRK-T, Hoffer-Q, Holladay-2, and Haigis) in predicting a target postoperative refraction ±1.0D in long eyes (axial length, 26.0 mm or more).

## Materials and Methods

### Study design

This was a prospective comparative study. After explaining the details of the study, we obtained written informed consent from all patients before enrollment. The study was approved by Mansoura University Hospital Trust Ethics Committee and was carried out in accordance with the Declaration of Helsinki (1989) of the World Medical Association.

### Patients

This study included 127 cataractous eyes of 87 patients with high myopia and axial lengths ≥ 26.0 mm attending the Mansoura Ophthalmic Center, Mansoura University, Egypt scheduled to undergo phacoemulsification. Patients with history of previous ocular surgery, posterior segment disorders, eventful cataract surgery, keratoconus, endothelial dystrophy, and glaucoma were excluded.

Preoperative examination included uncorrected and corrected distance visual acuity measurement, slit lamp biomicroscopy, Goldmann applanation tonometry, and indirect ophthalmoscopy after mydriasis. The axial length was measured using A-scan ultrasonic biometer (Compact II, Quantel Medical, Clermont, Ferrand, France). In all cases, the immersion ultrasound A-scan technique was used.

**Figure 1:**
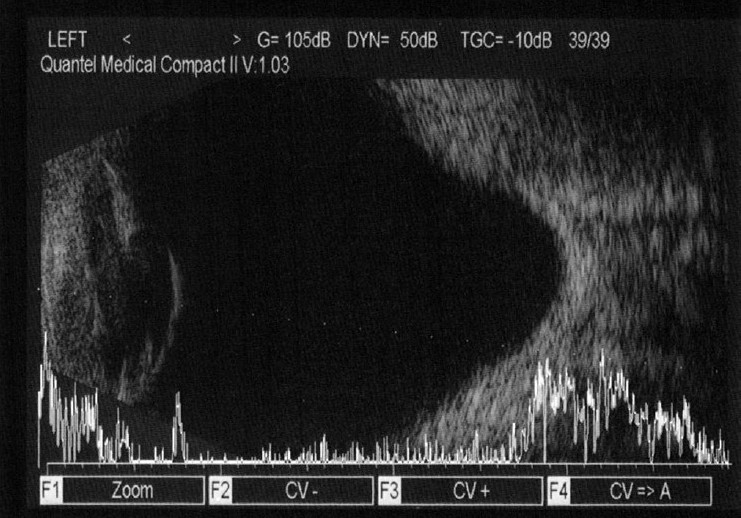
A/B-scan ultrasonography of an eye with posterior staphyloma in the study group

In eyes with dense cataract and poor fixation, combination A/B-scan method used. The B-scan axial length was determined by estimating an optical line through the eye (perpendicular to the cornea and lens) thus ensuring precise centration of the corneal peak. Therefore, in the presence of an eccentric staphyloma, the axial length chosen for biometry calculations did not necessarily represent the maximum geometric axial length of the eye [Figure 1]. As the B-scan allows visualization of the fovea, the retinal peak of the A-scan was aligned more exactly with it to avoid measurement of a staphyloma.

The corneal power (*k*) was measured using autokeratometer (NIDEK, autokeratometer KM 500, Gamgori, Japan). However, in some cases, computerized corneal topography was performed (Topo Shin Nippon CT 1000, Tokyo, Japan). Preoperative IOL power calculations were performed using SRK-T, Hoffer-Q, Holladay-2, and Haigis formulas in all patients. The surgeon’s goal in IOL power selection was a lens power that would yield a postoperative refraction ±1.0D. The IOL formula that provided a lens power with the above postoperative refraction was selected.

Phacoemulsification was performed through a sutureless 3.0–3.5 mm clear cornea incision, an anterior capsulorhexis of 5.2–5.5 mm, and a posterior chamber 3-piece hydrophobic foldable acrylic IOL (I-Medical, Weinheim, Germany) was implanted in the capsular bag. The patients were followed up for 4 months. Patients were examined 1 day postoperatively, then every week for the first month, then monthly for uncorrected and corrected distant visual acuity, autokeratometry, and fundus examination.

### Statistical analysis

All data were analysed with the SPSS version 15 (SPSS Inc., Chicago, IL, USA). Values were recorded as mean ±SD (standard deviation). A paired t-test was used for parametric comparison of the means. The mean axial length measurements and biometry errors were calculated. Deviation of the actual postoperative refraction from the assumed target preoperative refraction calculated and evaluated using a two-way analysis of variance (ANOVA). This spherical equivalent deviation was retrospectively compared to the different IOL formulas in this study. The confidence interval was 95% and *P* < 0.05 was considered statistically significant.

## Results

This study included 127 eyes of 87 patients (32 males and 55 females). Their age ranges from 42.5 to 63.0 years. The preoperative mean spherical equivalent refraction was -17.52 ± 4.01D, the mean corneal power was 44.68 ± 3.01, and the mean axial length was 31.71 ± 3.01 mm [Table 1]. Plus power IOLs were implanted in 60 eyes (plus-IOL power group), plano power IOL in 12 eyes, whereas minus power IOLs were implanted in 55 eyes (minus IOL power group).

**Table 1:** Preoperative data

Keratometric reading (D)	
Mean ± SD	44.68 ± 3.01
Range	40.05–55.14
Axial length measurement (mm)	
Mean ± SD	31.71 ± 2.85
Range	26.06–37.11
Anterior chamber depth (mm)	
Mean ± SD	3.66 ± 0.41
Range	3.15–4.75
Preoperative spherical equivalent (D)	
Mean ± SD	−17.52 ± 4.01
Range	−12.25–30.50

The percentage deviation of refractive outcomes from the calculated IOL power, in the plus and minus power groups, in each IOL formula group is seen in Tables 2 and 3. In the plus power group [Table 2], most postoperative refractions lay between 0.00 and −1.00*D* of the actual implanted IOL in all formula groups. On the other hand, in the minus power group [Table 3], most postoperative refractions were within +1.0 to +2.0D of the actual implanted IOL in all formula groups. However, Haigis formula showed the least deviation while SRK-T and other formulas showed a greater tendency toward hyperopia.

**Table 2:** Deviation of refractive outcome from assumed (target) refraction in plus-IOL power group

*Deviation in diopter*	*Diopters of deviation (%)*							
	*≤−3*	*−2*	*−1*	*0*	*+1*	*+2*	*+3*	**
Actual IOL implanted	10	18	28	32	7	5	0	%
SRK-T	15	10	31	38	6	0	0	%
Hoffer-Q	12	17	18	36	9	8	0	%
Holladay	4	18	21	30	6	15	6	%
Haigis	9	20	26	35	5	5	0	%

**Table 3:** Deviation of refractive outcome from assumed (target) refraction in minus-IOL power group

*Deviation in diopter*	*Diopters of deviation (%)*							
	*<−3*	*−2*	*−1*	*0*	*+1*	*+2*	*+3*	**D**
Actual IOL implanted	0	0	10	22	32	23	13	%
SRK-T	0	0	6	10	29	35	20	%
Hoffer-Q	0	3	6	17	30	26	18	%
Holladay-2	0	4	12	13	33	21	17	%
Haigis	0	11	20	28	21	12	8	%

**Figure 2:**
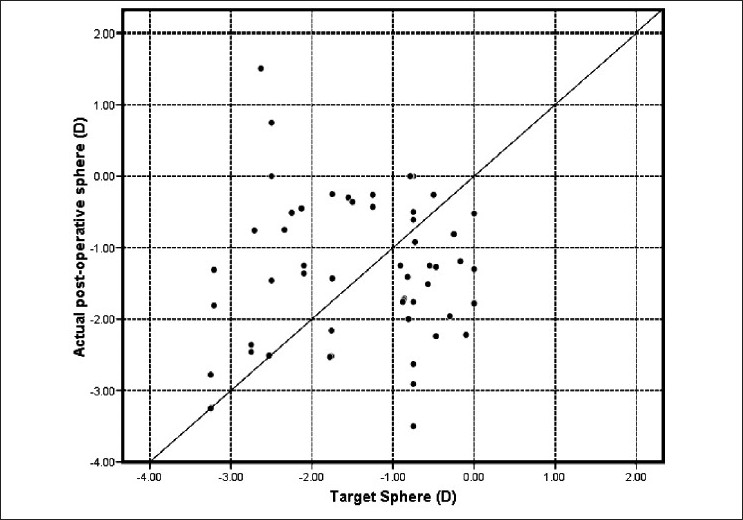
Target vs. actual postoprative sphere in plus power IOL group

**Figure 3:**
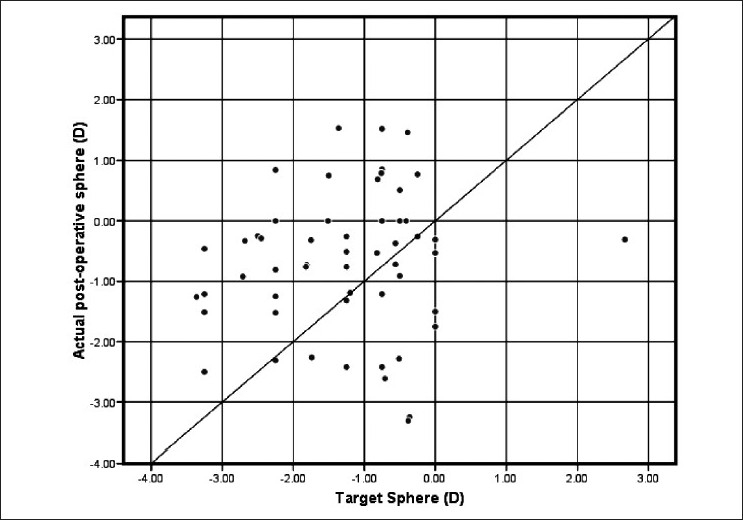
Target vs. actual postoprative sphere in minus power IOL group

**Figure 4:**
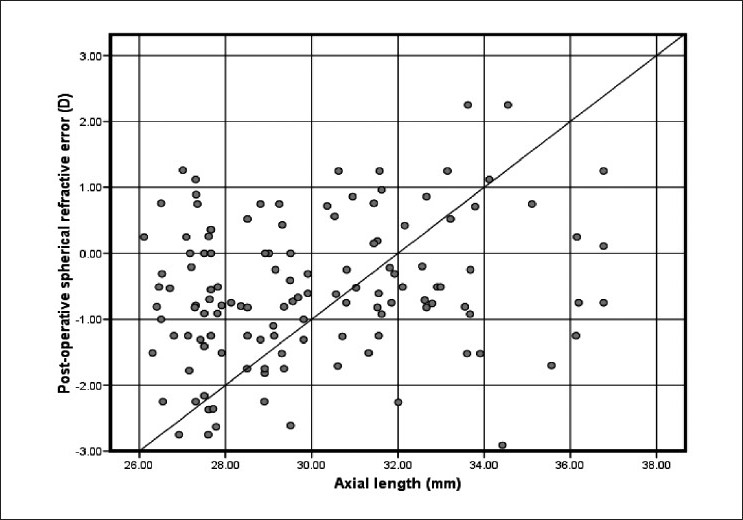
Correlation between axial length and postoperative spherical refractive error (D) in the study group

Figure 2shows a scatter plot of the target and actual postoperative refraction in plus power IOL group. Almost all postoperative refractions shown on the vertical axis are within the expected target refraction on the horizontal axis. The diagonal line represents the ideal correlation between the target and postoperative refraction. On the other hand, the scatter plot [Figure 3] of the minus power IOL group shows a positive bias to the biometry prediction. Figure 4 shows the correlation between axial length and postoperative spherical refractive error in the study group. It was noticed that hyperopic refraction is consistent with axial length more than 31.0 mm.

## Discussion

It has been suggested that IOL power calculation formulas work best for eyes with normal axial lengths. However, precise biometry prediction in extremely long eyes requiring concave lenses has always been difficult, although SRK-T is probably the most accurate formula and is now widely used. One biometry study including 40 eyes with axial lengths between 27 and 35 mm showed greater accuracy in high myopia with SRK-T formula than with SRK-2, Holladay, Hoffer-Q, or Binkhorst 2 formulas.[3–5]

In this study, we studied the refractive outcome of four different IOL power calculation formulas (SRK-T, Hoffer-Q, Holladay-2, and Haigis) in eyes with high myopia. The predictive capability of the four formulas is more or less satisfactory in the low-plus-power IOL group. The postoperative SE was ±1.0D of assumed refraction in 75%, 63%, 57%, and 66% of cases when using the four formulas preoperatively. In the minus IOL power group, there was an overall tendency toward hyperopia. The refractive outcome was within +1.0D in 45%, 53%, 58%, and 69%, respectively. The performance of Haigis and Holladay-2 are better than Hoffer-Q and SRK-T formulas.

Zaldivar *et al*.[6] reported that 92% of eyes were within ±1.0D when using SRK-T, 88% with Hoffer-Q in cases of plus power IOLs and in 54% with the SRK-T and Hoffer-Q formulas 63% with the Holladay-1 and 41% with Holladay-2 formulas in the case of minus power IOLs.

Petermeier and Szurman[7] reported the SRK-T, the Haigis, and the Holladay-1 formulas resulted in a mean hyperopic refractive error of +0.84D (SRK-T), +0.67D (Haigis), and +1.18D (Holladay-1), respectively, but within smaller range (SRK-T −0.55 ± 1.79D), Haigis (+0.04 ± 1.56D), Holladay-1 (-0.1 ± 2.07D). The mean axial length in this study was 32.35 mm (range, 29.22–36.51 mm).

The improved predictive capacity in eyes in which plus power were implanted is probably related to the improved accuracy of axial length measurements in these relatively shorter eyes.[6] The mean axial length was 29.61 ± 1.57 mm in patients receiving plus power IOLs and 33.32 ±1.51 mm in those receiving minus power IOLs.

The difficulties in IOL power calculations for longer eyes may be partly due to the anatomy of the posterior pole. The fovea is approximately 4.5 mm (3 disc diameter or 15°) from the center of the optic nerve. Holladay and Prager have performed high resolution B-scans with the innovative imaging system using horizontal sections through the optic nerve and measuring distance from the corneal vertex to a point 4.5 mm temporal to the center of the optic nerve.[8]

In eyes with axial lengths ≥ 30.0 mm, a posterior pole staphyloma temporal to the fovea was common, and the corneal vertex-fovea distance was approximately 0.5–1.5 mm, shorter than the distance from the corneal vertex to the bottom of the staphyloma, which is where the A-scan usually finds the perpendicular axis and records the axial length.[8] Immersion ultrasound A-scan reveals the anterior and posterior corneal peaks compared to a single peak with the applanation method. These two peaks of immersion technique may be a judge to the optical axis. Additionally, applanation method indents the cornea resulting in corneal compression between 0.14 and 0.47 mm. The immersion method measures the axial length from the anterior cornea to the inner-limiting membrane. The main disadvantage of immersion method is that misalignments may occur with the transducer probe, and the ultrasonic beam may not be perpendicular to the intraocular surface. This results in a jagged retinal peak or the absence of a posterior lens peak on the A-scan.

The use of optical biometry (IOL master) may further improve the accuracy by measuring axial length to the precise point of retinal fixation within a staphyloma.[9]

This study shows a similar refractive outcome as that reported by Zaldivar *et al*.[6] and MacLaren *et al*.[9] They showed a hyperopic tendency with minus power IOLs especially when SRK-T formula was used, and this tendency decreased with the Haigis formula.

Each formula has an A-constant associated with predicting the estimated lens power. The Holladay-1 formula uses a surgeon factor that is the distance between the iris plane and the power plane of the IOL, where the distance from the cornea to the iris plane is calculated as the dome height of the cornea. The Holladay-2 and Hoffer-Q formulas use an anterior chamber depth (ACD) constant which is the average distance between the power plane of the cornea and the IOL. The SRK-T formula uses the A-constant supplied by the manufacture of the IOL. The Haigis formula uses three constants: *a*0, *a*1, and *a*2. The *a*0 constant works in a similar manner as the constants for the other formulas. The *a*1 constant relates to the measured ACD (anterior corneal vertex to anterior vertex of crystalline), and the *a*2 constant to the measured axial length.

The problem with IOL power prediction using these formulas is that they rely on the axial length and central corneal power to predict the postoperative position of the IOL implant. These constants customized for the individual surgeon and IOL type to increase the accuracy in IOL power calculations. Holladay and Prager have shown that this often is not the case. Eyes with axial lengths of less than 20 mm often have large lenses, but otherwise completely normal anterior chamber anatomy.[8]

This basic assumption creates a mathematical limitation and is another reason why these formulas are not accurate over a wide range of axial lengths. Holladay-2 formula has done a good job of overcoming this limitation by using the measured ACD and several variables such as lens thickness and corneal diameter to better predict the final position of the IOL implant.[8,10–12]

Terzi *et al*.[13] and Haigis[14] concluded that optimization of lens constants improved the accuracy of IOL power calculation. Also, Petermeier *et al*.[15] found that with optimized constants, the SRK-T, Haigis, Hoffer-Q, and Holladay-1 formulas produced small deviation of postoperative refraction from target refraction.

Haigis formula differs in a very important way. It uses three constants: *a*0, *a*1 and *a*2 to calculate the effective lens position (*d*) where *d* = *a*0 + (*a*1 × ACD) + (*a*2 × AL). Therefore, rather than using a single number, the Haigis formula recommends IOL power based on three-variable (*a*0, *a*1, and *a*2) function.[16,17]

In eyes with high myopia, the performance of SRK-T, Hoffer-Q, Holladay-2, and Haigis formulas is good in low plus-powered IOLs implantation. However, a hyperopic refractive outcome is anticipated with minus power IOL implantation. Haigis formula is the best one when minus power IOL is implanted.

## References

[1] Badr IA, Hussain HM, Jabak M, Wagoner MD (1995). Extra-capsular cataract extraction with or without posterior chamber intraocular lenses in eyes with cataract and high myopia. Ophthalmology.

[2] Hill WE, Byrne SF, Points F (2004). Complex axial length measurements and Unusual IOL Power Calculations. Clinical Modules for Ophthalmologists.

[3] Garg A, Hoyos JE, Dementiev J (2005). Update on IOL power calculations formulas, in Mastering techniques of IOL power calculations.

[4] Sanders DR, Retzlaff JA, Kraff MC, Gimbel HV, Raanan MG (1990). Comparison of the SRK/T formula and other theoretical and regres-sion formulas. J Cataract Refract Surg.

[5] Kohnen S, Brauweiler P (1996). First results of cataract surgery and implantation of negative power intraocular lenses in highly myopic eyes. J Cataract Refract Surg.

[6] Zaldivar R, Shultz MC, Davidorf MJ, Holladay JT (2000). Intraocular lens power calculations in patients with extreme myopia. J Cataract Refract Surg.

[7] Petermeier K, Szurman P (2007). Accuracy of intraocular lens power calculation for the Acrysof® MA60MA in highly myopic patients. Book of abstracts. Book of abstracts.

[8] Holladay JT, Prager TC (1989). Accurate ultrasonic biometry in pseudophakia. Am J Ophthalmol.

[9] MacLaren RE, Sagoo MS, Restore M, Allan BD (2005). Biometry accuracy using zero – and negative-powered intraocular lenses. J Cataract Refract Surg.

[10] Smith LF, Stevens JD, Larkin F, Restori M (2001). Errors leading to unexpected pseudophakic ametropia. Eye.

[11] Kora Y, Koike M, Suzuki Y, Inatomi M, Fukado Y, Ozawa T (1991). Errors in IOL power calculations for axial high myopia. Ophthalmic Surg.

[12] Duffey RJ, Leaming D (2003). US trends in refractive sur-gery: 2002 ISRS survey. J Refract Surg.

[13] Terzi E, Wang L, Kohnen T (2009). Accuracy of modern intraocular lens power calculation formulas in refractive lens exchange for high myopia and high hyperopia. J Cataract Refract Surg.

[14] Haigis W (2009). Intraocular lens calculation in extreme myopia. J Cataract Refract Surg.

[15] Petermeier K, Gekeler F, Messias A, Spitzer MS, Haigis W, Szurman P (2009). Intraocular lens power calculation and optimized constants for highly myopic eyes. J Cataract Refract Surg.

[16] Haigis W, Shammas HJ (2003). Intraocular lens power calculations.

[17] Biometrie HW, Kampik A (1995). Yearbook of Ophthalmology 1995. Optics and Refraction.

